# Augmented TLR2 Expression on Monocytes in both Human Kawasaki Disease and a Mouse Model of Coronary Arteritis

**DOI:** 10.1371/journal.pone.0038635

**Published:** 2012-06-21

**Authors:** I-Chun Lin, Ho-Chang Kuo, Ying-Jui Lin, Feng-Shen Wang, Lin Wang, Shun-Chen Huang, Shao-Ju Chien, Chien-Fu Huang, Chih-Lu Wang, Hong-Ren Yu, Rong-Fu Chen, Kuender D. Yang

**Affiliations:** 1 Department of Pediatrics, Kaohsiung Chang Gung Memorial Hospital and the Graduate Institute of Clinical Medical Sciences, College of Medicine, Chang Gung University, Kaohsiung, Taiwan; 2 Department of Medical Research, Kaohsiung Chang Gung Memorial Hospital and the Graduate Institute of Clinical Medical Sciences, College of Medicine, Chang Gung University, Kaohsiung, Taiwan; 3 Department of Pathology, Kaohsiung Chang Gung Memorial Hospital and the Graduate Institute of Clinical Medical Sciences, College of Medicine, Chang Gung University, Kaohsiung, Taiwan; 4 Department of Pediatrics, Po-Jen Hospital, Kaohsiung, Taiwan; 5 Department of Medical Research, Show Chwan Memorial Hospital in Chang Bing, Changhua, Taiwan; Charité, Campus Benjamin Franklin, Germany

## Abstract

**Background:**

Kawasaki disease (KD) of unknown immunopathogenesis is an acute febrile systemic vasculitis and the leading cause of acquired heart diseases in childhood. To search for a better strategy for the prevention and treatment of KD, this study compared and validated human KD immunopathogenesis in a mouse model of *Lactobacillus casei* cell wall extract (LCWE)-induced coronary arteritis.

**Methods:**

Recruited subjects fulfilled the criteria of KD and were admitted for intravenous gamma globulin (IVIG) treatment at the Kaohsiung Chang Gung Memorial Hospital from 2001 to 2009. Blood samples from KD patients were collected before and after IVIG treatment, and cardiovascular abnormalities were examined by transthoracic echocardiography. Wild-type male BALB/c mice (4-week-old) were intraperitoneally injected with LCWE (1 mg/mL) to induce coronary arteritis. The induced immune response in mice was examined on days 1, 3, 7, and 14 post injections, and histopathology studies were performed on days 7 and 14.

**Results:**

Both human KD patients and LCWE-treated mice developed coronary arteritis, myocarditis, valvulitis, and pericarditis, as well as elevated plasma levels of interleukin (IL)-2, IL-6, IL-10, monocyte chemoattractant protein (MCP)-1, and tumor necrosis factor (TNF)-α in acute phase. Most of these proinflammatory cytokines declined to normal levels in mice, whereas normal levels were achieved in patients only after IVIG treatment, with a few exceptions. Toll-like receptor (TLR)-2, but not TLR4 surface enhancement on circulating CD14^+^ monocytes, was augmented in KD patients before IVIG treatment and in LCWE-treated mice, which declined in patients after IVIG treatment.

**Conclusion:**

This result suggests that that not only TLR2 augmentation on CD14^+^ monocytes might be an inflammatory marker for both human KD patients and LCWE-induced CAL mouse model but also this model is feasible for studying therapeutic strategies of coronary arteritis in human KD by modulating TLR2-mediated immune activation on CD14^+^ monocytes.

## Introduction

Kawasaki disease (KD) is a common cause of systemic vasculitis in young children, mostly affecting medium- and large-sized vessels. Coronary arteritis/coronary artery lesions (CALs), such as coronary artery ectasia and aneurysms, are the major devastating abnormalities associated with KD [1]–[2]. KD with CALs is one of the leading causes of acquired heart diseases in childhood [3]–[4]. Several clinical and pathological studies have indicated persistent vascular remodeling in these CALs [5]–[7]. Acute myocardial infarction or sudden death in young adults with childhood KD has been reported [8]–[9]. The current standard treatment with high-dose intravenous gamma globulin (IVIG, 2 g/kg) lowers the incidence of CALs from 25% to approximately 5% in cases of transient coronary ectasia and to 1% in cases of giant coronary aneurysms [10]–[11]. Nevertheless, resistance to the initial dose of IVIG is noted in 13–23% of KD patients with altered immune responses [12]–[13]. Since Dr. Kawasaki’s first report on KD in 1967, several immune alterations associated with KD have been reported [14]–[17]. The process by which systemic activation of the immune system progresses to leukocyte infiltration of coronary arteries and carditis in KD remains unclear.

Owing to the difficult access to coronary artery samples from human KD patients, several animal models, including mouse, rabbit, swine, and dog are employed to examine its immunopathogenesis [18]–[23]. *Lactobacillus casei* cell wall extract (LCWE) is commonly used to induce KD in animal models, inducing murine coronary arteritis similar to CALs associated with human KD [24]. Rosenkranz *et* *al.* [25] previously reported the importance of toll-like receptor (TLR)-2 signaling in LCWE-induced coronary arteritis by using TLR2^−/−^ knockout mice. In addition, we previously found that TLR2 augmentation on LCWE-treated RAW 264.7 cells–a murine monocyte/macrophage cell line–was positively correlated with LCWE-induced IL-6, MCP-1, and TNF-α upregulation [26]. However, it is possible that different host species may react differently, thereby posing a significant problem. For example, LCWE induces coronary arteritis in mice, whereas LCWE was reported to cause arthritis but not cardioangitis in rats [27]. Despite murine TLRs showing highly conserved homology with human TLRs, TLR2 regulation between mice and human differs considerably [28]. Therefore, we attempted to elucidate the involvement of TLRs in both human KD and LCWE-treated mice.

In this study, we further validated the cardiovascular pathologic lesions, immune alterations, and TLR2 expression in human KD patients in comparison with those in LCWE-treated mice. The common features of TLR2 augmentation on CD14^+^ monocytes, immune abnormalities and cardiovascular lesions suggest that not only TLR2 augmentation on CD14^+^ monocytes might be an inflammatory marker for both human KD patients and LCWE-induced CAL mouse model but also this model is suitable for investigating the immunopathogenesis of human KD, and it might lead to potential therapeutic strategies for the prevention or treatment of KD in patients with CALs.

## Results

### Coronary Arteritis, Myocarditis, Valvulitis, and Pericarditis in KD Patients

We studies 334 patients, including 223 boys (66.77%) and 111 girls (33.23%), at the mean age of 20.8±1.02 months, with a discharged diagnosis of KD from 2001 to 2009 at the Kaohsiung Chang Gung Memorial Hospital, Taiwan. There were no cases of mortality in this study. According to echocardiography studies, 118 patients (35.33%) had CAL formation, 7 patients had myocarditis (2.10%), 2 patients had pericarditis (0.60%), and 70 patients had valvulitis (20.96%) (Table 1).

**Table 1:** Cardiovascular abnormalities in Kawasaki disease (KD) patients and *Lactobacillus casei* cell wall extract (LCWE)-treated mice.

	Types of Lesions			
	Arteritis	Myocarditis	Pericarditis	Valvulitis
Human KD patients				
N = 334	118	7	2	70
	35.33%	2.10%	0.60%	20.96%
LCWE-treated Mice				
N = 23	20	12	7	5
	86.96%	52.17%	30.43%	21.74%

### Significant Increase in Systemic Pro-inflammatory Responses in KD Patients

**Figure 1:**
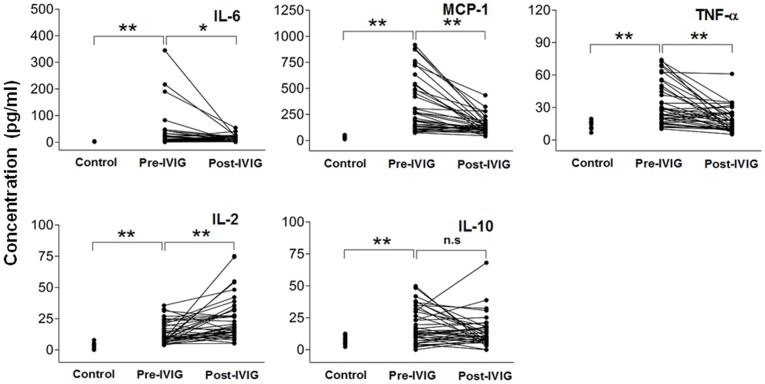
Immune abnormalities in human patients with Kawasaki disease (KD). Plasma levels of interleukin (IL)-6, monocyte chemoattractant protein (MCP)-1, tumor necrosis factor (TNF)-α, IL-2, and IL-10, assessed by Luminex technology, were significantly increased in KD patients before intravenous gamma globulin (Pre-IVIG), when compared to those in controls. IL-6, MCP-1, and TNF-α were significantly suppressed after IVIG treatment (Post-IVIG), but IL-2 and IL-10 responded differently. Values are expressed as mean±SEM; n = 39 in paired KD patients, n = 15 in control patients. *P<0.05; **P<0.01.

The plasma levels of IL-2, IL-6, IL-10, MCP-1 and TNF-α were studied in 39 human KD patients before (Pre-IVIG) and after IVIG (Post-IVIG) treatment in pairs and in 15 control patients (Figure 1). Plasma levels of IL-6 were significantly higher in KD patients before IVIG treatment (33.96±10.92 pg/ml) than those in the control patients (2.49±0.18 pg/ml) and those after IVIG treatment (10.85±1.85 pg/ml) (*P* = 0.006, *P* = 0.029, respectively). Similarly, MCP-1 levels were higher in KD patients before IVIG (328.26±41.32 pg/ml) than those in control patients (32.73±3.48 pg/ml) and those after IVIG (142.34±12.58 pg/ml) (*P*<0.001, *P*<0.001, respectively). TNF-α levels were higher in KD patients before IVIG (33.18±3.08 pg/ml) than those in control patients (13.83±1.08 pg/ml) and those after IVIG (18.28±1.77 pg/ml) (*P*<0.001, *P*<0.001, respectively). Interestingly, IL-2 levels were higher in KD patients before IVIG (13.52±1.38 pg/ml) when compared to control levels (3.03±0.62 pg/ml) (*P*<0.001), but remained significantly elevated even after IVIG (24.95±2.78 pg/ml) (*P*<0.001). IL-10 levels were higher in KD patients before IVIG (18.31±2.26 pg/ml) when compared to control levels (7.61±0.84 pg/ml) (*P*<0.001) and remained higher after IVIG (14.16±1.99 pg/ml).

### Significant Augmentation of TLR2 Surface Expression on Peripheral Monocytes in KD Patients

**Figure 2:**
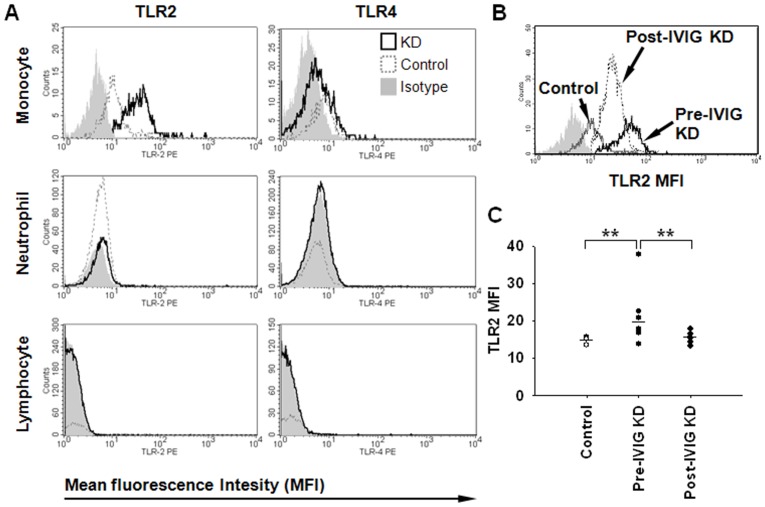
TLR2 and TLR4 surface expression on peripheral blood leukocytes (PBLs) in human KD patients. (A) Representative histograms showed surface expression of TLR2 and TLR4 by mean fluorescence intensity (MFI) on peripheral monocytes, neutrophils, and lymphocytes in KD patients before IVIG treatment and control patients. (B) The representative histogram displayed surface expression of TLR2 by MFI on peripheral CD14^+^ monocytes in human KD patients before and after IVIG treatment as well as those in control patients. (C) TLR2 expression over CD14^+^ monocytes are expressed as mean±SEM by MFI. n = 6 in controls; n = 12 Pre-IVIG; n = 8 Post-IVIG. **P<0.01.

Flow cytometry on PBLs using mean fluorescence intensity (MFI) was performed to analyze the surface expression of TLR2 and TLR4 in KD patients. The percentages of human monocytes expressing surface TLR2 varied widely, ranging from 7.76% to 70.08% in control patients (n = 6, controls), 4.2% to 91.61% in KD patients before IVIG (n = 12, Pre-IVIG KD), and 3.78% to 57.25% in KD patients after IVIG treatment (n = 8, Post-IVIG KD) (Figure 2A and B). Only a few neutrophils and even fewer lymphocytes expressed TLR2 on their surface. Except for some monocytes with low intensity expression of surface TLR4, there were few TLR4-positive circulating neutrophils and lymphocytes in KD patients and control patients. Furthermore, TLR2 enhancement over CD14^+^ monocytes was significantly augmented in KD patients before IVIG treatment compared to that in control patients (*P* = 0.004), which significantly decreased in patients after IVIG treatment (*P* = 0.005) (Figure 2C).

### Immune Alterations in LCWE-treated Mice

**Figure 3:**
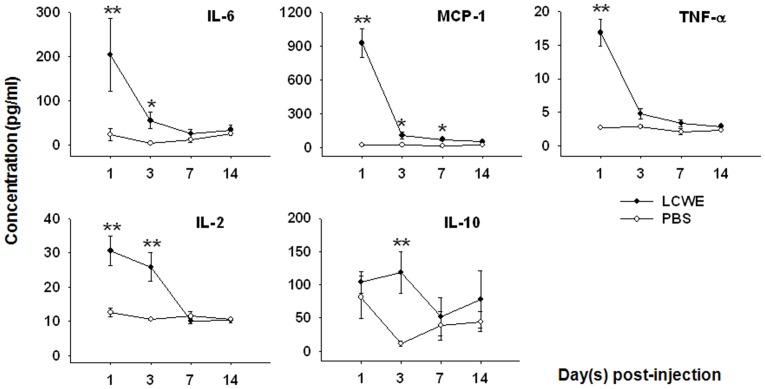
Immune abnormalities in the *Lactobacillus casei* cell wall extract (LCWE)-treated mice. Plasma levels of IL-6, MCP-1, TNF-α, IL-2, and IL-10 were assessed by Luminex technology. IL-2, IL-6, MCP-1 and TNF-α were significantly increased early after LCWE stimulation and spontaneously declined to basal levels during days 3 and 7 post injection. In contrast, IL-10 was significantly elevated at day 3 post injection. Values are expressed as mean±SEM; n = 9 in LCWE-treated mice; n≥7 in PBS-treated mice, per time point for all, except n = 5 for IL-2 per group per time point. *P<0.05; **P<0.01.

Next, IL-2, IL-6, IL-10, MCP-1, and TNF-α levels were measured in the plasma from mice sacrificed after LCWE treatment for 1, 3, 7, and 14 days (n = 9 except n = 5 for IL-2, per time point) (Figure 3). Plasma IL-6 and IL-2 levels in LCWE-treated mice on days 1 and 3 were significantly higher than those in controls (n≥7, per time point) (*P* = 0.003, *P = *0.037, respectively in IL-6; *P* = 0.009, *P* = 0.007, respectively in IL-2). On days 7 and 14, both plasma IL-6 and IL-2 levels in LCWE-treated mice declined and were not statistically different from those in controls. Similarly, LCWE-treated mice exhibited considerably higher plasma MCP-1 levels than control levels on post-injection days 1, 3, and 7 (*P*<0.001, *P = *0.024, *P = *0.011, respectively). On day 14, plasma MCP-1 levels in LCWE-treated mice were higher, but not significantly higher, than those in controls. Further, the plasma level of TNF-α in LCWE-treated mice on day 1 was higher than those in controls (*P*<0.001). On days 3, 7 and 14, plasma levels of TNF-α in LCWE-treated mice declined, and later resembling the levels measured in controls. The plasma IL-10 level was significantly increased in LCWE-treated mice on day 3 (*P = *0.003), but spontaneous recovery to the basal level was observed at later time points.

### Significant Augmentation of TLR2 Expression on Monocytes of LCWE-treated Mice

**Figure 4:**
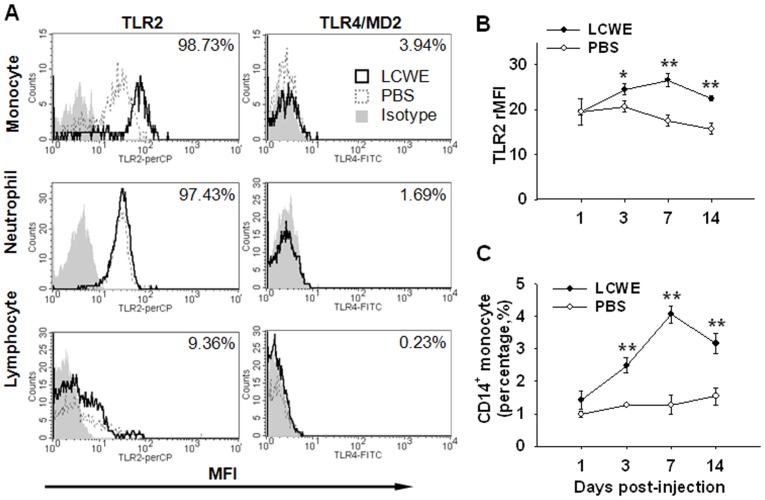
TLR2 and TLR4/MD2 surface expression in LCWE-treated mice. (A) Representative histograms showed surface expression of TLR2 and TLR4/MD2 by MFI on monocytes, neutrophils, and lymphocytes in the LCWE-treated mice and PBS-treated control mice on post-injection day 7. The numbers labeled over the right upper quadrants indicated the representative percentage of TLR2^+^ or TLR4/MD2^+^ cells, corresponding to each cell subpopulation. (B) The surface expression of TLR2 on circulating CD14^+^ monocytes were analyzed by relative TLR2 MFI (rMFI = TLR2 MFI/isotype MFI) at the indicated time points post-injection. (C) Circulating CD14^+^ monocytes were significantly increased in LCWE-treated mice on days 3, 7, and 14 post injections. Values are expressed as mean±SME, n = 7 mice/group per time point; *P<0.05; **P<0.01 versus PBS controls.

In LCWE-treated or PBS-treated mice, the majority of circulating monocytes and neutrophils, as well as a few lymphocytes, expressed surface TLR2 with stronger intensity observed on monocytes than on neutrophils (Figure 4A). In contrast, TLR4/MD2 surface expression on monocytes, neutrophils, and lymphocytes was much lower than TLR2 expression, regardless of LCWE treatment. To confirm the TLR2 expression on monocytes in LCWE-treated mice, we used dual staining of CD14 and TLR2 to track the kinetic expression of TLR2 on CD14^+^ monocytes on days 1, 3, 7, and 14 post injection (n = 7, per time point). The results indicated that TLR2 surface expression on CD14^+^ monocytes significantly increased on days 3, 7 and 14 in LCWE-treated mice compared to that in the control mice (*P = *0.035, *P* = 0.004, *P = *0.002, respectively) (Figure 4B). In addition, it was found that CD14^+^ monocytes significantly increased in LCWE-treated mice when compared to control CD14^+^ monocytes after 3 days (*P = *0.004 on day 3, *P = *0.002 on day 7, *P* = 0.006 on day 14) (Figure 4C).

### Cardiovascular Lesions in LCWE-treated Mice

**Figure 5:**
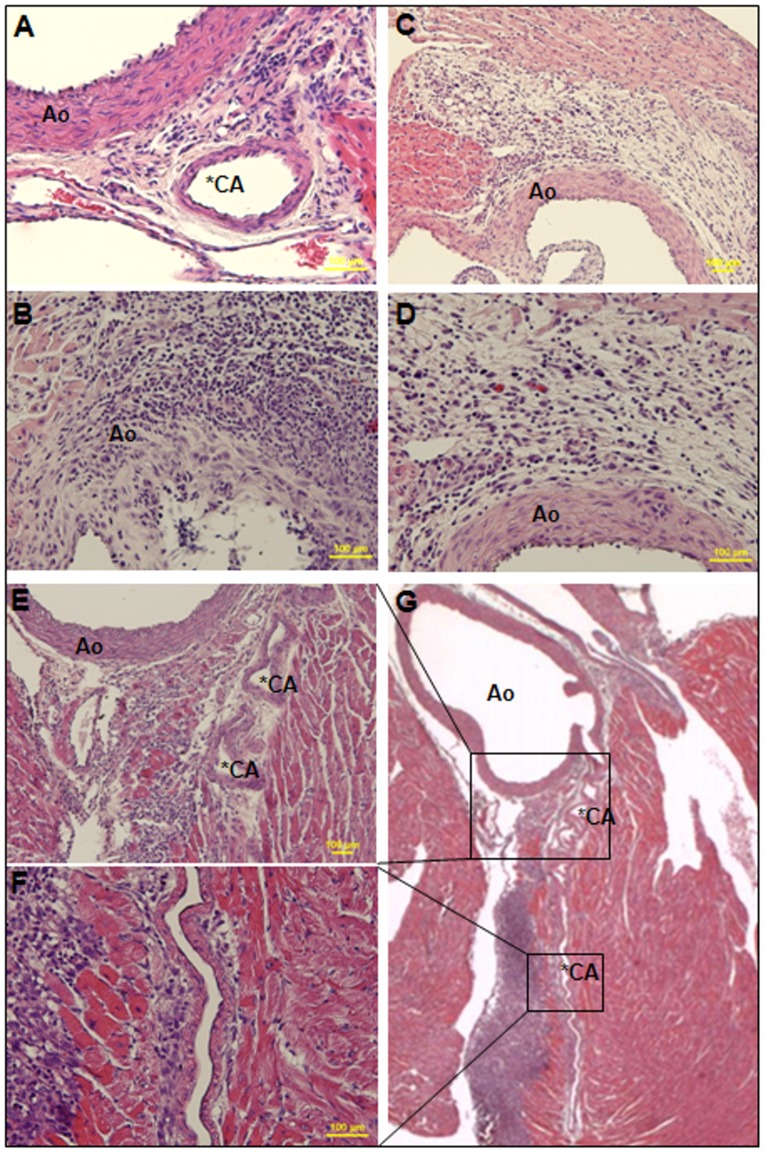
Histopathology of cardiac tissues in LCWE-treated mice. (A–G) Arteritis developed as infiltration of mononuclear cells and neutrophils in the perivascular/adventitial regions of the aortic vessel/aortic valve (Ao) and coronary artery (CA) (D is the higher magnification of C). Myocarditis (B, D, F and G), valvulitis (B), and pericarditis (G) were complicated with the infiltration invading into the myocardium, aortic valve, and pericardium, respectively. (E, F) Higher magnification of black boxes on the serial section next to (G) showed infiltration around and along the vessel walls of the CA and myocardium in detail. Original magnification: ×200 in (A, B, D and F), ×100 in (C, E) and ×10 in (G). Each yellow bar  = 100 µm. Black asterisks indicate the lumen of the CA.

Heart tissues from 23 mice treated with LCWE (LCWE-treated mice) were subjected to H&E examination on days 7 (n = 11) and 14 (n = 12) post injection and compared to those of the controls. None of the controls treated with PBS (n = 18) developed any pathological lesion on days 7 and 14. The overall induction rate of arteritis was 86.96% (20/23); myocarditis, 52.17% (12/23); pericarditis, 30.43% (7/23); and valvulitis, 21.74% (5/23) (Table 1). Arteritis was most frequently associated with some mononuclear cell and neutrophil infiltration in the perivascular/adventitial region of the aorta and coronary artery on day 7 (Figure 5). Some mice showed more prominent infiltration invading into the myocardium (Figure 5C), aortic valve (Figure 5B), or pericardium (Figure 5G and F). Furthermore, no inflammatory lesion was observed in the liver, lung, kidney, or spleen.

## Discussion

The results of this study demonstrate the similar features of monocyte TLR2 augmentation, immune abnormalities and cardiovascular lesions in human KD patients and LCWE-treated mice. KD, a childhood cardiovascular disease, leads to chronic and persistent vascular inflammation of coronary arteries until adulthood. Since the importance of TLR2 in LCWE-mediated arteritis, the aim of our study was to assess the involvement of TLR2 on CD14^+^ monocytes in both human KD and LCWE-induced arteritis mice, and to further validate the translational application of this mouse model for investigating human KD.

TLR2 augmentation on CD14^+^ monocytes is a distinct feature shared by KD patients and LCWE-treated mice, although TLR2 expression is a little dissimilar between human and mice. In contrast, no differential expression of TLR4 was observed between KD patients and control patients, or between LCWE-treated mice and PBS-control mice. Meanwhile, an increase in circulating CD14^+^ monocytes was found in LCWE-treated mice, a result compatible with those of our clinical study [29] showing that persistent monocytosis after IVIG therapy was associated with CALs in KD patients. We previously found that TLR2 augmentation on RAW 264.7 cells might be indicative of strong inflammation of monocytes/macrophages due to this augmentation was positively correlated with LCWE-induced IL-6, MCP-1, and TNF-α upregulation [26]. Meanwhile, these upregulation of proinflammatory cytokines/chemokines could be suppressed *in vitro* by using TLR2 but not TLR4 neutralizing antibody (Figure S1). Taken together, TLR2 augmentation on monocytes may serve an inflammatory marker in both human KD patients and the LCWE-induced arteritis mouse model, and TLR2 may play a crucial role in the immunopathogenesis of human KD as in the LCWE-induced arteritis mouse model [25].

Among 334 recruited KD patients, approximately one-third and one-fifth of them developed coronary arteritis and valvulitis, respectively. Very few KD patients had myocarditis (2.1%) and pericarditis (0.6%). The rate of CALs, valvulitis, myocarditis, and pericarditis/pericardial effusion had been reported ranging from 5% to 46%, 4.2% to 53%, 15% to 65%, and 30%, respectively, revealed by different diagnostic modalities [30]–[32]. It is noteworthy that these 4 types of cardiovascular lesions were also observed in LCWE-treated mice. However, the lower incidence of myocarditis and pericarditis in our KD patients may partly result from the method of diagnosis. All instances of pericarditis in mice were diagnosed by histopathology, and pericarditis always occurred in association with myocarditis invading into the pericardium. While isolated pericardial effusion was not visually seen in LCWE-treated mice, it was detected in a few KD patients by echocardiography. Moreover, subtle/occult myocarditis would not cause significant myocardial depression and impaired ejection fraction detectable by echocardiography. This subtlety might cause an underestimation of the incidence of myocarditis in KD patients, especially for the milder form of myocarditis. Taken together, LCWE could efficiently induce mice to develop the same cardiovascular lesions as those in human KD patients. This mimicry warrants the investigation of the underlying mechanism of the LCWE-induced arteritis mouse model and its experimental application in translational study.

In order to survey the immunopathogenesis of KD, some immune responses were assessed both in KD patients and LCWE-treated mice. Marked elevation of IL-6, MCP-1, and TNF-α suggested a robust systemic inflammation in the early stages in both human disease and the mouse model. In human KD patients, these 3 cytokines significantly declined after IVIG treatment but remained statistically higher than those in controls, though samples from KD patients without IVIG treatment were unavailable. CALs in KD patients often occurred before the systemic inflammation completely subsided. In contrast, a spontaneous recovery of these 3 cytokines occurred in LCWE-treated mice, nearly reaching basal levels without any treatment during 3 and 7 days post injection, which was then followed by the development of CALs. LCWE-treated mice exhibited subsequent increases in cardiac IL-6, MCP-1, and TNF-α [26]. Apparently, a spatial and temporal differentiation between systemic and cardiac inflammation were observed in LCWE-induced arteritis mice [26]. These results may imply the same differentiation in human KD and thus, highlight the necessity of this animal model to study KD.

Alterations in systemic IL-2 and IL-10 differ between human KD patients and LCWE-treated mice. IL-2 significantly increased after IVIG treatment in humans, whereas LCWE-treated mice showed a spontaneous decline in plasma level of IL-2 at 7 days post injection. IL-10 levels before and after IVIG treatment did not differ in human KD, whereas elevation in IL-10 increased in the early phase after LCWE treatment and returned to basal levels thereafter. IL-2 is a leukocytotrophic cytokine, leading to T-cell activation. The role of T cell activation in pathogenesis of human KD remains an area of intense research. Persistent elevation of systemic IL-2 even 3–7 days after IVIG treatment might be indicative of continuing T cell activation. However, a spontaneous recovery of systemic IL-2, like other cytokines, could not account for the local cardiac inflammation in the mouse model. In agreement with a study performed by Schulte *et* *al.* [33], we also found that the involvement of T cells contributed to the local cardiac inflammation, as assessed by immunohistochemistry, showing CD3- and CD4-positive cells around the cardiac infiltration, concomitant with increased mRNA levels of interferon-γ and IL-17A (data not shown).

In conclusion, we addressed the involvement of CD14^+^ monocytes and their TLR2 enhancement as well as dynamic immune responses and cardiovascular lesions in both human KD patients and LCWE-treated mice. These shared features suggest that TLR2 augmentation may be a candidate indicator of CD14^+^ monocyte activation in human KD patients and LCWE-treated mice. In addition, the LCWE-induced arteritis mouse model is feasible for studying the immunopathogenesis of coronary arteritis in human KD and for determining methods for the prevention of coronary arteritis in human KD by blocking TLR2-mediated immune activation on CD14^+^ monocytes.

## Materials and Methods

### Ethics Statement

"The study protocol involving human participants conformed to the Declaration of Helsinki and was approved by the Institute Review Board of Kaohsiung Chang Gung Memorial Hospital (Permit number: 96-0279B, 96-0359B, and 99-4059B). All human subjects gave permission through the informed written consent form from the next of kin, or guardians on the behalf of the minors/children participants involved in our study. All clinical investigations were conducted according to the principles expressed in the Declaration of Helsinki.

All animal experiments were performed in accordance with legislation on the protection of animals and were approved (Permit number: 2009071301, 2007032602) by the animal care committee at Chang Gung Memorial Hospital". All procedures were performed under anesthesia, and all efforts were made to minimize suffering.

### Patients and Samples

All subjects studied were children diagnosed with KD, who were admitted for IVIG treatment at the Kaohsiung Chang Gung Memorial Hospital from 2001 to 2009. Blood samples from control patients admitted for upper and/or lower respiratory tract infections were also included for comparison. The echocardiography reports were reviewed retrospectively. CAL/arteritis was defined as a coronary artery having an internal diameter of at least 3 mm (4 mm if the subject was over the age of 5 years) or a segment with an internal diameter at least 1.5 times larger than that of an adjacent segment by echocardiogram [34]–[35]. Myocarditis was defined as a left ventricle ejection fraction of ≤54%, and pericarditis was defined as acute phase pericardial effusion. Valvulitis was defined as (1) aortic valve regurgitation and mitral valve regurgitation at acute and regressed during follow-up and/or (2) tricuspid valve regurgitation greater than moderate grade at the acute phase that regressed during follow-up.

### LCWE Preparation

LCWE was prepared as previously described [26]. Briefly, cells of *Lactobacillus casei* (ATCC 11578, Bioresource Collection and Research Center, Taiwan) were first cultured in Lactobacillus MRS broth (Difco, Detroit, MI) at 37°C. After harvested, the cells were treated overnight with 4% SDS (Sigma-Aldrich, St. Louis, MO), followed by sequentially incubated with 250 µg/ml RNase, DNaseI, and trypsin (Sigma-Aldrich). The final pellet was then sonicated (5 g packed wet weight in 15 ml PBS) for 2 hours at a pulse setting of 9 second pulse/5 second pause at 20 kHz frequency (Vibra Cell™, Sonics & Materials Inc., Newtown, CT). Following 1-hour centrifugation at 20,000×*g*, the supernatant concentration was determined on the basis of its rhamnose content by using a phenol-sulfuric acid colorimetric assay [36]. The endotoxin concentration of this preparation was <1.5 pg/µg (data not shown), as determined by the Limulus amoebocyte lysate assay (Associates of Cape Cod Inc., East Falmouth, MA).

### Mice and Animal Model of Coronary Arteritis

Wild-type BALB/c mice, purchased from the National Laboratory Animal Center, Taiwan, were used in this study. Four-week-old male mice were intraperitoneally injected with either 1 ml of PBS alone as controls or PBS containing 1 mg of LCWE [24], [26] as treatment mice. Heart tissue sections were obtained for examination of coronary arteritis and myocarditis on post-injection days 7 and 14 because infiltrates began in the adventitia of coronary arteries as early as day 3–7 post-LCWE injection.

### Cytokine Measurement by the Luminex^100^ System

Cytokine levels in the plasma of KD patients and mice were assessed using a commercially available Human and Mouse Cytokine/Chemokine Milliplex™ MAP kit (Millipore Corp., Billerica, MA) as follows: interleukin (IL)-2, IL-6, IL-10, monocyte chemoattractant protein (MCP)-1, and tumor necrosis factor (TNF)-α. This assay has been designed for the quantitative measurement of multiple cytokines/chemokines in a single well using as little as 25 µl of the sample, followed by detection using Luminex^100^ Flowmetrix systems (Luminex Corp., Austin, TX).

### Flow Cytometric Analysis

For analyzing the surface expression of TLR2 and TLR4, we conducted double-stain of CD14/TLR2 and CD14/TLR4 in human whole blood, and triple-stain of CD14/TLR2/TLR4 in murine samples. Each staining reaction was incubated for 30 minutes with the following primary antibodies and their corresponding isotypes (all from eBioscience, San Diego, CA) at 4°C as follows: fluorescein isothiocyanate (FITC)-conjugated anti-human CD14, Phycoerythrin (PE)-conjugated anti-human TLR2, PE-conjugated anti-human TLR4, allophycocyanin-labeled anti-mouse CD14, biotin-conjugated anti-mouse TLR2 (subsequently being labeled with streptavidin-peridinin-chlorophyll protein complex), and PE-conjugated anti-mouse TLR4/MD2. After the staining procedure, peripheral blood leukocytes (PBLs) were isolated and then resuspended in 200 µl PBS containing 1% paraformaldehyde for the flow cytometry analysis using FACSCalibur^®^ (Becton Dickinson, San Jose, CA). The percent expression of CD14 was assessed using quadrants in the two-color mode. The surface expression of TLR2 was calculated as the relative mean fluorescence intensity (rMFI) normalized by the fluorescence intensity of the isotype control in mice.

### Histopathology

Murine cardiac tissues were fixed in formalin, and embedded in paraffin. Coronary arteries and the nearby aortic valve were serially identified (4 µm-thickness sections), and stained with hematoxylin and eosin (H&E). Blinded microscopic assessment by 2 pathologists was performed to identify coronary/aorta arteritis, and/or myocarditis, depending upon the presence of inflammatory infiltrates and damage to the cardiomyocytes and vessel walls as well as valvulitis and pericarditis. In addition, the lungs, spleens, livers, and kidneys were also excised for histopathological examination.

### Statistical Analysis

Data are presented as mean±SEM. The biochemical parameters of the murine samples and human TLR2 expression were compared using Mann-Whitey *U* test. A standard 2-tailed *t*-test was used for analyzing human plasma data and paired *t*-test was used in comparison of pre-IVIG data to post-IVIG data. A *P* value of <0.05 determined using SPSS was considered statistically significant.

## Supporting Information

Figure S1 **Effects of neutralizing anti-TLR2 (TLR2B) and anti-TLR4 (TLR4) antibodies on LCWE-treated RAW 264.7 cells.** IL-6, MCP-1, and TNF-α mRNA expression were studied on RAW 264.7 cells. Data, presented as mean±SEM, were derived from 3 independent experiments and normalized to β-actin mRNA levels. *P<0.05 by Mann-Whitney *U* test.
(DOC)
