# Supplementary material for: Augmented TLR2 Expression on Monocytes in both Human Kawasaki Disease and a Mouse Model of Coronary Arteritis
Source: PLoS One. 2012 Jun 21;7(6):e38635. doi: 10.1371/journal.pone.0038635 (PMC3380902; doi:10.1371/journal.pone.0038635)
Supplement: Figure S1 — Effects of neutralizing anti-TLR2 (TLR2B) and anti-TLR4 (TLR4) antibodies on LCWE-treated RAW 264.7 cells. IL-6, MCP-1, and TNF-α mRNA expression were studied on RAW 264.7 cells. Data, presented as mean±SEM, were derived from 3 independent experiments and normalized to β-actin mRNA levels. *P<0.05 by Mann-Whitney U test. (DOC) [file pone.0038635.s001.doc]

**Supporting Information**

**Figure S1. Effects of neutralizing anti-TLR2 (TLR2B) and anti-TLR4 (TLR4) antibodies on LCWE-treated RAW 264.7 cells.** IL-6, MCP-1, and TNF-α mRNA expression were studied on RAW 264.7 cells. Data, presented as mean ± SEM, were derived from 3 independent experiments and normalized to β-actin mRNA levels. **P* < 0.05 by Mann-Whitney *U* test.
